# Spontaneous Cerebro-Spinal Fluid Rhinorrhoea Caused by Sustained Intracranial Hypertension

**DOI:** 10.7759/cureus.24441

**Published:** 2022-04-24

**Authors:** Moataz Younis, Mohammed Adly, Mohamed Yousry, Adel Zahran, Amr Elmoheen

**Affiliations:** 1 Emergency Department, Hamad Medical Corporation, Doha, QAT; 2 Medicine, Qatar University, Doha, QAT

**Keywords:** spontaneous csf leak, spontaneous csf rhinorrhea, cerebrospinal fluid rhinorrhea, csf leakage, idiopathic intracranial hypertension (iih)

## Abstract

Cerebrospinal Fluid (CSF) leakage results from a defect in the skull base, which communicates the subarachnoid space with the nasal cavity. The most common cause of CSF leakage is traumatic, and non-traumatic causes are less common. This case report illustrates a case of a woman who presented to the emergency department with clear fluid pouring from her nose for three weeks with a fever. The patient had pneumococcal meningitis and Idiopathic Intracranial Hypertension (ICH) seven years ago. Computed Tomography (CT) sinuses showed the defect seen on the right side of the cribriform plate, and the Magnetic Resonant Imaging (MRI) confirmed the CSF leakage. The CSF leakage was diagnosed by positive B transferrin.

This case highlights a rare condition that needs early detection and treatment to prevent complications such as ascending meningitis.

## Introduction

The cerebrospinal fluid (CSF) is a glucose-rich and clear-protein liquid in the subarachnoid space of the central nervous system. It is a physiologic fluid whose function is to protect the brain and maintain intracranial pressure (ICP). The CSF is produced at the choroid plexus. The choroid plexus circulates and turns over 140 mL of CSF daily. Severe craniomaxillofacial trauma destroys the meninges, which may lead to leakage of the CSF from the subarachnoid space. It is important to note that post-traumatic leaks manifest in at least 1% to 3% of closed traumatic brain injuries in adults. It is also worth mentioning that 80% to 90% of CSF leaks are attributed to head injuries [1,2]. CSF leakages occur when a defect connects the subarachnoid space and other spaces. This happens mainly during meningeal damage. The primary cause of CSF leak is a structural distortion due to craniofacial trauma. This makes up 80% of the leaks. 16% of CSF leaks are attributed to iatrogenic causes, while the remaining 4% include congenital defects and spontaneous leak defects [3].

A traumatic CSF leak increases the risk of meningitis. The risk may present with high morbidity and, in some cases, mortality depending on the cause of the CSF leak and the site of the leak. Traumatic leakage of the cerebrospinal fluid is highly harmful and is accompanied by varying complications, including bacterial meningitis [4]. Conventional treatment involves the intravenous administration of antibiotics as well as repair of the dural defect in the event of a definite injury. CSF leaks must be detected early, as early detection and treatment determine the patient’s prognosis. The clinician's decision on whether to engage in surgical intervention or to just observe depends on the cause of the leak, as well as the site and timing [5].

## Case presentation

A 38-year-old female patient presented to the emergency department with clear fluid pouring from her right nostril (rhinorrhea) for three weeks. She has had a history of Idiopathic Intracranial Hypertension for 7 years and was treated with a spinal tap and medications to reduce the intracranial pressure. She had a history of pneumococcal meningitis with consequent hearing impairment five months ago.

The rhinorrhea was increasing with bending forward. She had a history of fever for one day. She did not suffer from vomiting or dizziness. She denied exposure to head trauma or assault, visual deficits, or other complaints.

Physical examination of the patient showed that she was vitally stable with a temperature of 36.6°C, a pulse of 86 beats per minute, a respiratory rate of 18 breaths per minute, and O2 saturation of 97% on room air. She was fully conscious, had no neurological deficit, and her pupils were equal and reactive bilaterally. She had ecchymosis in the left inferior-orbital area. Her cardiovascular and respiratory system examination was normal. Her abdomen was lax and had no tenderness.

Investigations

Her blood investigations showed mild elevation of the White Blood Cells (WBCs), with predominant neutrophils (ANC-absolute neutrophil count) and mildly elevated C-reactive protein (CRP); however, other blood tests were unremarkable (Table 1). The rhinorrhea fluid was positive for the Beta-2 transferrin assay.

**Table 1:** Laboratory investigation results

Test	Result	Normal range
White Blood Cells (WBCs)	11.7 × 10^9^/L	4.5-11.0 × 10^9^/L
Absolute Neutrophil Count (ANC)	8.3 × 10^9^/L	2.0-7.0 × 10^9^/L
Lymphocyte	2.2 × 10^9^/L	1.0-3.0 × 10^9^/L
Hemoglobin (Hgb)	11.7 g/dl	11.6-15 g/dl
Platelets	444 × 10^9^/L	150-450 × 10^9^/L
Urea	2.9 mmol/L	2.1-8.5 mmol/L
Creatinine	60 µmol/L	61.9-114.9 µmol/L
Sodium (Na)	137 mmol/L	135-145 mmol/L
Potassium (K)	4.7 mmol/L	3.5-5.0 mmol/L
Chloride (Cl)	121 mmol/L	96-110 mmol/L
Bicarbonate (HCO3)	12.0 mmol/L	21.8-26.2 mmol/L
C-reactive protein (CRP)	14.6 mg/L	Less than 10 mg/L

**Figure 1:**
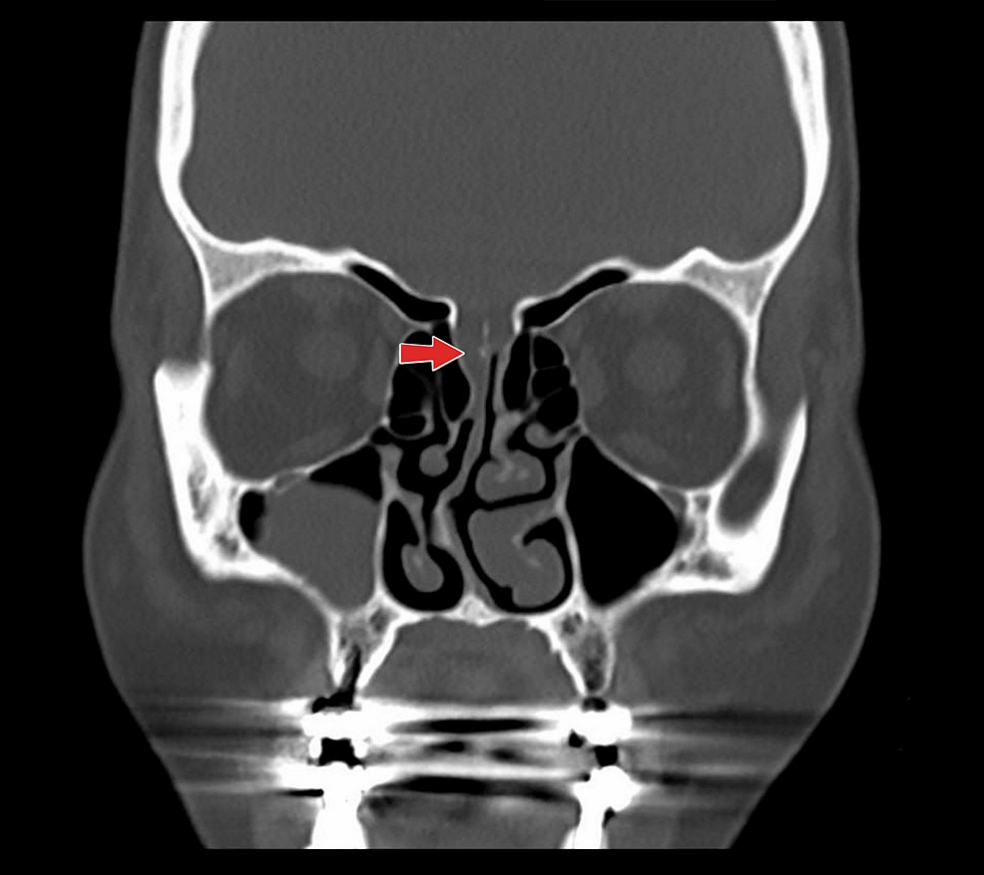
Computed Tomography (CT) sinuses showing a probable defect seen in the right side of the cribriform plate (red arrow), with fluid density seen at the upper nasal cavity.

Computed Tomography (CT) of sinuses showed a probable defect seen on the right side of the cribriform plate (Figure 1), with fluid density seen in the upper nasal cavity. It also showed a nasal septal deviation toward the right side with a bony spur abutting the right inferior nasal turbinate and mucosal hypertrophy of the left middle and inferior terminate.

**Figure 2:**
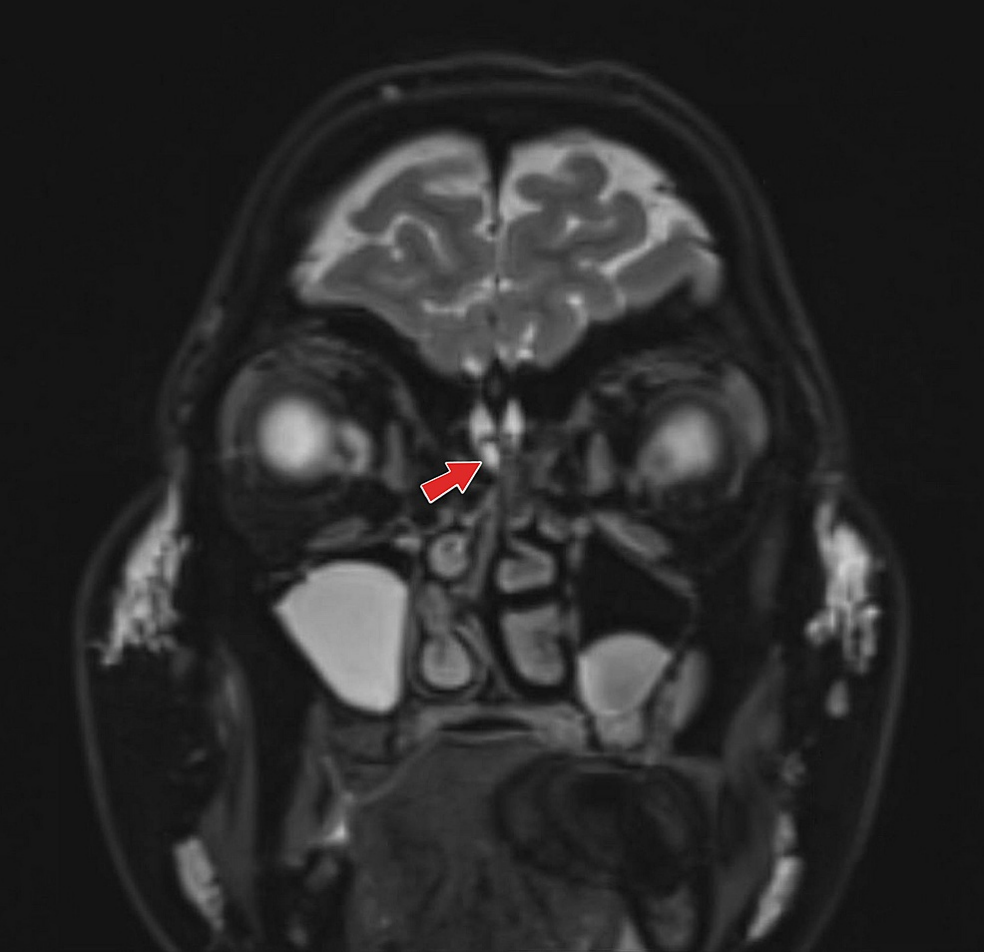
Magnetic Resonant Imaging (MRI) head with contrast showed a small amount of high signal intensity on the T2-weighted image (T2WI) seen infero-medial to the right olfactory bulb (red arrow), which suspected Cerebrospinal Fluid (CSF) leakage.

Magnetic Resonant Imaging (MRI) head with contrast showed evidence of idiopathic intracranial hypertension. There was a small amount of high signal intensity on the T2-weighted image (T2WI) seen infero-medial to the right olfactory bulb (Figure 2), which suspects CSF leakage.

Differential diagnosis

The patient was initially thought to have an upper respiratory tract infection or sinusitis. The ecchymosis in her left inferior-orbital area raised suspicion of a bony defect. The CT sinuses confirmed a spontaneous CSF leak at the skull base, and the MRI head confirmed the diagnosis. There was no suspicion of head trauma or domestic violence.

Treatment and follow-up

The neurosurgery team recommended doing an endoscopic repair of the skull-base defect, but the patient preferred not to do the operation. The patient was discharged home with her regular medications with regular follow-up in the outpatient neurology clinic.

## Discussion

CSF leakage results from a defect in the skull base, which communicates the subarachnoid space with the nasal cavity. Non-traumatic spontaneous CSF leakage is rare and accounts for less than 5% of all reported cases [6]. Spontaneous cerebrospinal fluid rhinorrhoea is associated with intracranial hypertension and elevated body mass index (BMI) [7].

The pathogenesis of CSF leakage is not fully understood, but results from past studies suggest that prolonged intracranial hypertension (ICH) may cause defects in the skull base. These skull base defects alongside ICH may lead to dura mater herniation. The dura mater herniates into the bony defects, causing the dura mater to get weak and increasing its susceptibility to dural tears [8]. In the same way, obesity triggers a rise in intra-abdominal pressure, causing the elevation of the diaphragm and ultimately leading to increased cardiac and pleural pressures. This decreases venous return to the heart from the brain and causes ICH [9]. The patient in the present case had idiopathic intracranial hypertension, as suggestive by the old MRI head with contrast which showed tortuous optic nerves with prominent perioptic CSF spaces flattening of the optic nerves and an empty Sella Turcica.

The presence of CSF is best detected by testing for beta-2 trace protein or beta-2 transferrin [10, 11]. Beta-2 transferrin is a primary component of CSF, the vitreous humor, and the perilymphatic fluid with 95% specificity and 100% sensitivity [10]. In the case of our patient, blood investigation for Beta-2 transferrin turned out positive. A study [9] was unable to assess beta-2 transferrin testing the use of comparative glucose concentrations of the draining fluid. Glucose in secretions indicates the presence of CSF. However, this method is not recommended due to low diagnostic specificity and sensitivity as well as false-negative results in the event of bacterial contamination or false positives when dealing with diabetic patients [10]. Detecting glucose in CSF rhinorrhoea may not be used on its own to diagnose leakage of the CSF and calls for detailed clinical and radiographic evidence [11].

High-resolution MRI and CT scans are most reliable when differentiating spontaneous CSF rhinorrhoea from nonspontaneous rhinorrhoea. Both techniques assist in localizing leaks, mostly when associated with tumors or fractures of surrounding bones. They do not, however, demonstrate the leakage.

MR/CT cisternography is the gold standard for CSF leak detection as it can identify the size of the leak and the quantity and location. However, the procedure is invasive and may not be necessary if the diagnosis is supported by imaging results (MRI and CT) and clinical presentation [10]. In the case of our patient, a CT scan of the sinuses showed a probable defect on the right side of the cribriform plate with fluid density seen in the upper nasal cavity. A conservative approach may be employed at the initial stage of the treatment. If this fails, then surgery is done. Conservative treatment of cerebrospinal fluid leakage involves the use of acetazolamide followed by extended bed rest with head elevation, which may cause the intracranial pressure to decrease [12]. As employed in our case, surgical intervention may involve either an extracranial/endoscopic approach or an intracranial approach. The intracranial approach has a high risk of morbidity and about 20% to 40% failure rates. On the other hand, the endoscopic approach has a 90% - 100% success rate and less risk of morbidity [5]. A study that examined 193 cases of CSF leaks over a 21-year timeline found that endoscopic repair had an overall success rate of 98%. This, together with low morbidity, has confirmed endoscopic repair as the gold standard for the repair of cerebrospinal fluid leaks [13].

Early detection and treatment play important roles in preventing complications associated with CSF leakages, such as intracranial sepsis, meningitis, and abscesses. These complications are all associated with high mortality rates. However, a successful surgical repair and an uneventful post-operative period guarantee a favorable prognosis.

## Conclusions

Spontaneous non-traumatic CSF leakage is a rare condition characterized by elevated ICH and BMI. The Beta-2 transferrin test serves as the gold standard for detecting and diagnosing CSF leaks in secretions. MR/CT cisternography is the most efficient imaging test as it identifies the leak with precision. Early detection and treatment prevent complications such as ascending meningitis.
